# From radiology to a world-in-crisis: Rolf Sievert and the re-orientation of the International Commission on Radiation Protection in the post-war period

**DOI:** 10.1017/mdh.2025.10038

**Published:** 2026-10

**Authors:** Aske Hennelund Nielsen

**Affiliations:** Chair of Science, Technology and Gender Studies, Friedrich-Alexander-Universität Erlangen-Nürnberg, Erlangen, Germany

**Keywords:** Rolf Sievert, Diplo-scientific actors, International organisations, ICRP, Post-war, Radiation protection

## Abstract

With this paper, I suggest focusing on *diplo-scientific actors* as a fruitful approach to study how certain actors have helped to shape international organisations through their diplomatic activities and scientific practices. Using the example of the Swedish medical physicist Rolf Sievert, I show how Sievert’s personality and preferences came to decisively shape the post-war trajectory of the International Commission on Radiation Protection (ICRP) and more broadly the international landscape of radiation protection. In the inter-war period, Sievert was engaged in the ICRP and its mother organisation, the International Radiological Congress (IRC), dealing with the interwoven questions of how to formulate international radiation standards and radiation protection initiatives. Following the Second World War, Sievert became concerned with the proliferation of nuclear technologies and the spread of radioactive fallout following nuclear test bombings. Because of this concern, Sievert set out to separate the ICRP from the IRC and form a new, independent group that could deal with the dangers of the new ‘Nuclear Age’. While Sievert was ultimately unsuccessful, his attempts would decisively change the purview and trajectory of the ICRP. This had large ramifications, as the ICRP continues to be the prime international organisation on radiological and medical radiation protection, formulating recommendations that are used by international, regional, national, and local groups, informing epistemic judgements on radiation research.

## Introduction

The atomic attacks on Hiroshima and Nagasaki changed the world in innumerable ways, with long-lasting effects on society and technology still felt to this day. One area that perhaps was influenced more than others was the field of radiation protection. While the pre-war period had seen controversies and cases of obvious neglect, fraud, and harm – most noticeably the case of the Radium Girls in the 1920s – the majority of the population were not exposed to any radiation risks.^1^ This all changed after 1945. The proliferation of nuclear technologies in various sectors was spurred on also by industrial initiatives that meant more workers were engaged with or working in radioactive environments.^2^ At the same time, the issues of nuclear fallout were becoming a growing concern, as the increase of radiation in the atmosphere following nuclear bomb testing became progressively more obvious.^3^ One scientific and medical community anticipated these developments with much trepidation, namely, the medical radiologists who had been using x-rays and ionising radiation in their medical practice since the end of the nineteenth century. As some of the prominent, early actors dealing with radiation protection, these medical doctors and health physicists were intimately familiar with the risks of overexposure to radiation – having also seen many of their own colleagues perish because of it.^4^ The fact that more and larger populations were being exposed to radiation could not help but be a cause for concern.

Prior to the outbreak of the Second World War, the main international organisations dealing with the issues of radiation standardisation and nuclear research in medicine had been the radiological committees of the International Radiological Congress (IRC), namely the International Commission on Radiation Protection (ICRP), and the International Commission on Radiation Units and Measurements (ICRUM) both founded in 1928 in Stockholm. Both groups had attracted some of the most prominent medical-scientific actors of their time, like Lauriston S. Taylor of the US National Bureau of Standards, Gioacchino Failla of the Memorial Hospital New York, William Mayneord of the Royal Marsden Hospital in London and Herman Holthusen of the Allgemeines Krankenhaus St. Georg in Hamburg, to develop and, in a diplomatic fashion, negotiate the standardisation of radiation units and radiation protection.^5^ One of the most prominent of these actors was Rolf Maximilian Sievert (1896–1966). Sievert was a physicist with a career spanning decades in his native Sweden and on the international scene, who played a decisive and influential role in the international development of radiation protection in both the pre- and post-war periods.

In this article, I use Sievert as an example to illustrate how a medical and scientific actor can impact, influence, and steer the development of international organisations. The emerging science diplomacy scholarship has emphasised the co-production of science and diplomacy,^6^ highlighting how science can be informed by diplomatic negotiations and vice versa, diplomatic activities can be influenced by scientific practices. I wish to contribute to this emerging scholarly field by focusing on what Nielsen and Knudsen have called *the extraordinary human factor*,^7^ drawing attention to the importance of individual actors who have played a strong and decisive role through their intertwined diplomatic negotiations and scientific practices. Indeed, this type of actor might appropriately be termed *diplo-scientific actor.* These actors have played an instrumental role in several contexts, but in this article, I wish to focus on their role in shaping international organisations through their scientific practices and diplomatic activities. International organisations are here of particular interest, as they construct underlying infrastructures of epistemic claims and accepted knowledge on for instance scientific standards, recommendations, literature, and materials also for national, regional, and local groups.^8^

The approach I am arguing for here is not entirely novel, as other scholars have also dealt with scientists engaged in diplomatic activities through a biographic approach.^9^ Previous research has also highlighted the interconnected nature between science and diplomacy. For instance, Latour and Woolgar in *Laboratory Life* have highlighted that the social dynamics of the laboratory space are reminiscent of other social settings, with scientists also competing with rival laboratories in order to gain prestige within their epistemic circles.^10^ Preste has shown how science and politics are deeply intertwined processes,^11^ while other recent research has highlighted how scientific and technical conferences have combined diplomacy and science at different levels.^12^ I therefore wish to build on these insights into how scientists interact in (diplomatic) social settings, by focusing specifically on the interconnected activities of certain actors’ *science* and *diplomacy* in international contexts. This approach complements other recent research that has also focused on specific actors in the intersections of science and diplomacy.^13^

Inherent in the study of diplo-scientific actors is also a biographic approach – and I am therefore also inspired by the field within the history of science called *scientific biography.* ^14^ I here draw on the insights that biographical aspects of a scientist’s experience, personality, preferences, and dislikes can influence and shape their scientific work in various ways. My purpose here is therefore not to give an authoritative biography of Sievert’s life and work in relation to his scientific output, rather, I focus on certain periods of Sievert’s life to showcase how his experiences and preferences came to impact his scientific and diplomatic approach and ultimately shape the current order of international organisations dealing with radiation protection.

Sievert is here an instructive example because of his long and influential career in the domestic and international setting. Sievert was head of the Radiophysics Laboratory of the Swedish radiological centre Radiumhemmet [The Home of Radium] in Stockholm for over forty years.^15^ Some of Sievert’s international credentials include being the chairman of the United Nations Scientific Committee on the Effects of Radiation Protection (UNSCEAR) from 1958 to 1960, as well as chairman of the ICRP from 1956 to 1962.^16^ In 1979, thirteen years after his death, Sievert received one of the highest honours a scientist can receive when a unit of radiation was named after him.^17^ Sievert was a highly esteemed researcher and scientific actor within the field of radiation protection and commanded authority through his forceful presence,^18^ however, despite this, Sievert and his impact on the field of radiology, radiation protection, and radiation standardisation and units has not been wholly studied in the international literature. Reasons for this oversight are related to the fact that much of the material related to Sievert and his activities is in Swedish and that the archival materials are spread over several different institutions in Sweden.

An important and central aspect of not only Sievert’s career, but also radiation protection as a scientific process, is the medical, radiological practice that Sievert and his contemporaries acted within. While Sievert was not trained as a physician, he spent most of his career deeply engaged within the medical system in Sweden and internationally. The interlocked issues of radiation protection and radiation standardisation did not arise first and foremost from scientific discussions; rather, they arose from practical, medical concerns of how to apply X-rays and radium to treat patients and limit danger to personnel. Not only were these standards important so as to ensure that patients, but especially the healthcare workers, were not exposed to excessive amounts of radiation, but the formalisation of international standards also allowed the radiological clinics to carry out inter-country comparisons to determine better treatment options.^19^

These concerns of standardisation and protection did not diminish after the Second World War, on the contrary. With the proliferation of radioisotopes in various new sectors including the formation of new medical specialities like nuclear medicine that arose out of this dissemination,^20^ the role of the ICRP in formulating medical protection was becoming even more present. However, due to Sievert’s growing concerns that radiation protection had to be expanded to include not just medical doctors and patients, but also larger populations, the role of the IRCP also grew manyfold to include groups outside of medicine. Much of this anxiety felt by Sievert should be traced back to the discourse of health in the radiological community. Having spent decades discussing standardisation and radiation protection, the radiological community felt that they had both the expertise and responsibility to enact changes to try and contain any health risks caused by radiation. This also illustrates the coupling of expert knowledge – in this case on medical uses of radiation – to more general societal concerns of health and risk in the twentieth century and beyond.

The article is therefore structured around two periods of Sievert’s career, namely, the last years before the Second World War and the immediate post-war period, ending around 1960. I have based my account on archival sources related to Sievert and Radiumhemmet located at the Karolinska Hospital Archive, as well as various published sources related to the IRCP and the ICRUM. In the conclusion, I offer some thoughts on how diplo-scientific actors can influence international organisations and what ramifications this can have.

## Standardisation efforts of the inter-war period, 1925–1939

**Figure 1.**
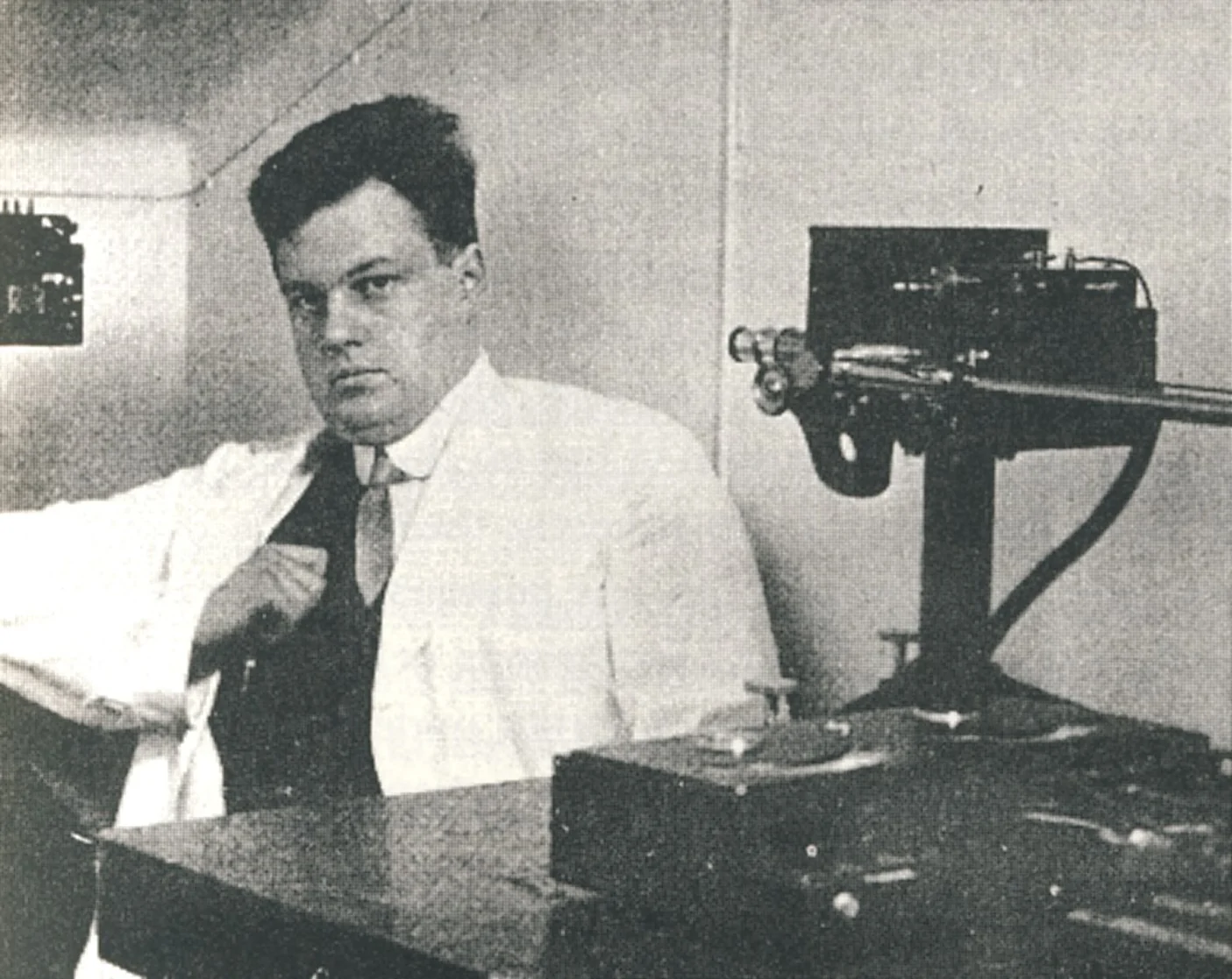
Rolf Sievert in the Radiophysics Laboratory of Radiumhemmet [The Home of Radium] in the mid-1920s. The picture is in the public domain and taken from: <https://commons.wikimedia.org/wiki/File:Rolf_Sievert.jpg *>*

The first International Congress of Radiology (IRC) was held in London in 1925, with the second congress held in 1928 in Stockholm. At the Stockholm meeting, the ICRUM^21^ and the ICRP^22^ were founded, also marking Rolf Sievert’s entrance onto the international scene. Sievert was a member of the ICRUM from the beginning and was named the first president of the ICRP at its foundation in 1928, and would continue to be involved in both groups until the 1960s (Figure 1).^23^

During the next congress held in Paris in 1931, the ‘r-unit’ or ‘röntgen-unit’ that had been suggested at the 1928 congress was voted in as the standard unit for radiation measurement by the ICRUM, becoming the *de facto* standard for medical radiology in the inter-war period. A set of radiation protection standards of British origin was also accepted in Paris by the ICRP, likewise proposed in Stockholm in 1928. These proposals were satisfactory compromises for the members of the ICRP and the ICRUM, signifying an end to the national disagreements of the 1920s.^24^ However, this balance would be upset at the 1937 congress in Chicago. The American physicist Lauriston S. Taylor, who, like Sievert, would play a large role in both the ICRP and the ICRUM throughout most of his career, would try to recruit Sievert to his and the Americans’ side in a scientific dispute with the British delegation. Prior to the 1937 congress, the British radiologists were planning to expand the r-unit to include not only x-rays, as was the original intention of this standard but also gamma-radiation. Taylor and the US radiologists strongly opposed this, arguing that the addition of gamma radiation did not have any practical, medical use, and sought to curry Sievert’s favour against the British proposal.^25^ Taylor was successful, leading to a compromise between the forceful insistence of the British Delegation and the resistance of the Americans and Swedes. At the 1937 congress, aspects of the British proposal for the r-unit were incorporated, however, they were explicitly labelled ‘provisional’, to be determined and finalised at the next congress in Hamburg in 1940, which never came to pass because of the outbreak of the Second World War.

Following the 1937 congress, the issues of the standardisation of radioactive units in radiology were therefore in upheaval. Different groups, notably the British against the Americans and Swedes, were in disagreement on what constituted the most scientifically valid and medically practical x-ray and gamma unit. However, an unexpected third party would present itself to Sievert, offering new opportunities. In September 1938, A. Establier^26^ of the League of Nations’ (LoN) International Institute of Intellectual Cooperation would write a letter to Sievert, asking him to take part in a conference in Groningen from 28 to 29 October 1938 on ‘Measurements of ionizing radiations’.^27^ Besides Sievert, eleven prominent radiologists had been invited, relevant here being Gioacchino Failla of the New York Memorial Hospital, William Valentine Mayneord of the Royal Marsden Hospital in the UK, Hermann Holthusen of the Allgemeinen Krankenhauses St. George in Hamburg, and Louis Harold Gray of the Mount Vernon Hospital in London.^28^ Establier requested that Sievert prepare a full paper on ‘Quantitative determination of the ionizing radiation on outer and in-depth in biological systems’ and hoped that Sievert would serve as chair for the conference.

This was not the first time that the LoN had dealt with the question of radiation and radioactive units. Already in 1931, the organisation had commissioned a group to study the biological effects of radiation and to suggest protection measures.^29^ The German radiologists Hermann Wintz and Walther Rump had been tasked to write the report on the subject, titled *Protective Measures Against Dangers Resulting from the Use of Radium and Roentgen.* While the report received praise by medical journals around its publication in August 1931,^30^ it does not seem to have informed the standardisation process of the ICRP or the ICRUM in any meaningful way. Previous to this, Marie Curie had also served as a scientific authority in the International Institute of Intellectual Cooperation, the same office that was now contacting Sievert, from 1921 to 1934.^31^

Sievert did not get a chance to respond to Establier’s request before he was contacted by Holthusen, one of Sievert’s confidants. In Holthusen’s letter, he expressed surprise and concern.^32^ Firstly, as Germany was not a member of the LoN, Holthusen and the other invited Germans, Friedrich and Jaeger, would likely not be able to attend the conference, potentially cutting off Germany from shaping any decisions made in Groningen.^33^ Secondly, as the IRC Congresses of 1925 and 1928 had already discussed the issue detailed in the invitation from Establier, Holthusen saw little point in repeating these debates at the LoN conference. Thirdly, Holthusen feared that the more ‘politically charged atmosphere’ of the LoN would be detrimental to the work of x-ray standardisation. Holthusen speculated that perhaps some radiologists or physicists from the ICR had encouraged the LoN to set up this meeting, in order to discuss the ‘… urgent questions of the measurement of radiation calmly, outside the hectic tempo of the IRC’.^34^ Holthusen implored Sievert to make sure that the LoN meeting would be kept to just that, a singular meeting.

While the invitation from the LoN also came as a surprise to Sievert, he saw more opportunities to exploit this meeting than Holthusen did.^35^ Sievert was by nature a deeply efficient person who had a strong sense of organisational prowess.^36^ As such, Sievert also harboured a strong aversion to the large and, at least to him, often unwieldy ICRUM committee, which already at the 1925 IRC consisted of twenty-four members from fourteen countries.^37^ Sievert’s preference leaned towards smaller committees that would be able to carry out quick action. The Groningen meeting therefore presented an opportunity for Sievert to discuss the question of how to determine a proper medical dosage anew, with people he considered suited for the task – namely Gray, Failla, Holthusen, and himself – prior to the next International Congress in 1940.^38^

Sievert therefore not only responded to Establier in September 1938, praising the initiative of the LoN, but also requested several revisions.^39^ The large group of eleven radiologists was untenable, with Sievert instead suggesting a smaller working group of Failla, Gray, Holthusen, Lacassagne, and himself. Sievert stressed that it was critical for Holthusen to join owing to his pioneering scientific work within the radiological field, and further suggested streamlining the conference by having the participants submit short summaries on the topic of radiation dosage prior to the meeting.

Sievert’s hopes for the LoN to gather a small working group were to be dashed by a long and drawn-out process over the next several months. Establier contacted Sievert in early October, stating that the LoN was not planning to convene a permanent commission, but to bring together a group of prominent experts to make a statement on the current status of the topic of radiation standards.^40^ In the same letter, Establier also informed Sievert that the conference had been postponed to February 1939. Sievert responded maintaining his previous suggestions for the meeting, and stating that he did not intend to attend the meeting if his revisions were not followed.^41^ They would not be.

At this junction, it is relevant to highlight some aspects of Sievert’s personality and quirks, as they pertain to the process of the LoN Groningen conference and to his later post-war engagement. Sievert notoriously hated traveling, to the point where he would often buy tickets for both air and ship travel, deciding on the day which form of transportation he preferred – or perhaps he disliked the least.^42^ Bo Lindell, who was Sievert’s close collaborator for many years, gives an impression of Sievert’s phobia: ‘Sievert saw trips as hazardous undertakings. Ships could sink, aircraft fall and trains crash’.^43^ Sievert therefore only undertook journeys he considered absolutely necessary – and increasingly the Groningen conference seemed an unnecessary meeting to him. Sievert’s work process should also be considered here. Weinberger has highlighted that Sievert took great steps in preparing meticulous proposals, which he hoped would give him an advantage over his competitors.^44^ As mentioned, Sievert hoped to do exactly this at the Groningen conference; by gathering a small group of like-minded scientists, Sievert hoped to prepare new suggestions for new radiation dosages that he could present at the 1940 IRC, which he believed would give him an advantage over the other groups at the Congress.

After several delays and much back-and-forth, the Groningen conference was finally set to run from 30 May to 1 June 1939, however, continued issues regarding the attendees at the conference would further sour Sievert’s mood. In May 1939, Failla informed Sievert that neither he nor his research assistant Edith Quimby could make it from the USA to Europe.^45^ Quimby had been Failla’s close collaborator for many years,^46^ and Sievert valued both her and Failla’s scientific contributions highly. Writing to Establier, Sievert decried that Failla, Quimby, and Holthusen would be unable to attend the Groningen conference, stating that he was only interested in participating in the meeting if ‘some valuable work can be done’ and that the meeting should be postponed,^47^ with little success. In the end, as neither Failla, Quimby, nor Holthusen could attend the Groningen conference, Sievert decided not to attend either. In a short letter to Establier on 26 May just four days before the start of the conference, Sievert stated in no uncertain terms that: ‘I think you know that in my opinion the conference can hardly be successful unless prof. Holthusen and dr. [sic] Failla are present.’^48^

For Sievert, the outcome of the Groningen conference was a failure, as it did not bring him closer to reformulating the medical radiation dosage to his liking. Despite Sievert’s frustrations, he still left the door open for the LoN to assemble a working group on radiation standardisation at a later date. The breakout of the Second World War in Europe in September 1939 made any such arrangements impossible, and prevented international activities in the IRC for almost a decade. However, Sievert’s frustration with the LoN system and the process for the Groningen conference would become relevant again in the post-war period.

## Towards new horizons and a new Commission, 1949–1953

Following the end of the Second World War, the activities of the IRC and its committees, the ICRUM and the ICRP were being restarted towards 1949. Like other international organisations that had started major projects that had been interrupted by the war,^49^ the ICRUM and the ICRP were ready to take on the issues left over from 1939. However, a new reality had set in following the war. The advent of nuclear technologies in several new sectors, the imminent constructions of atomic power plants, as well as the dangers of fallout following nuclear weapons testing signified the dawn of the ‘Atomic Age’,^50^ and with it, many new radiation protection challenges.

At this junction, in the immediate post-war period, a shift was occurring where radiation protection stopped being primarily a concern of radiological and medical professionals, to being a more generalisable concern. The ICRP and the ICRUM continued to formulate standards and protection initiatives for medical practice. However, the fact that radiation was now being extended to not only other medical sectors – including the burgeoning field of nuclear medicine – but also other industrial and scientific arenas and the potential risk of nuclear fallout, meant that more and diverse groups were being exposed to radiation than ever before. This development also tied in with another critical aspect in the post-war period, namely, the changing landscape of international organisations. As Boudia has shown, the practices of existing and new international institutions changed radically after the Second World War: ‘[The] Organization of international regulations after 1950 took the form of a subtle game between various bodies, each one constantly having to redefine its role and its scope of action in relation to other institutions and prevailing conditions.’^51^ This was no different for radiation protection, where several international organisations were carving out territories for themselves.^52^ Animated by the proliferation of the nuclear industry and radioactive fallout, international organisations like the World Health Organization (WHO), the International Labour Organization (ILO), the World Meteorological Organization (WMO), and the International Atomic Energy Agency (IAEA) were taking an increasing interest in radiation protection for their specialised areas and also competing with each other.^53^ Sievert was in the middle of these winds of change. With the experience of the LoN, the presence of the new international juggernaut of the United Nations (UN), and the dangers of the atomic age, the question of radiation standardisation and protection still lay heavy on his mind.^54^

The relative isolation caused by the Second World War of the different national communities concerned with radiation protection and radiation standards had meant that the inter-war relations had been shaken up. Notably, the USA, Canada and Britain had found common ground during a meeting in 1949,^55^ and, at the suggestion of Failla, the three parties had agreed to set a weekly limit of exposure of 0.3 röntgen for ‘workers’,^56^ meaning all individuals who could be expected to be exposed to radiation as part of their workday, be that in a medical, industrial or scientific setting. This new grouping would also make its presence felt in the international arena of the IRC. At the first post-war congress of the IRC in London in 1950, the USA, UK, and Canadian group strongly argued that their suggestion of a dosage of 0.3 röntgen should be introduced as a new international standard, with Sievert and Jaeger of Germany arguing for a significantly lower dose of 0.1 röntgen per week. Sievert and Jaeger were, however, stonewalled by the Anglo-Saxon alliance. The new recommendations of the ICRP were published in *The British Journal of Radiology* in January 1951, using the USA, UK, and Canadian 0.3 röntgen standard for workers dealing with radiation.^57^ The recommendations did however note that the advent of the nuclear age had ‘… greatly increased the number and scope of potential hazards’, necessitating continuous revisions of these standards and that it was not possible to ‘… make firm recommendations regarding the maximum permissible amounts of radioactive isotopes that may be taken into, or retained in the body’.^58^

Around this time in the early 1950s, Sievert seems to have come to the conclusion that the ICRP was no longer able to perform its duty satisfactorily. The inter-war medical and radiological focus of the ICRP was becoming too narrow in view of the expansion of the nuclear industry and the release of radioactivity in the atmosphere, and the presence of new groupings in the IRC like the USA, UK, and Canadian alliance who opposed Sievert’s suggestion, no doubt contributed to his frustration. In 1952, Sievert therefore started work to re-orient the ICRP to face the challenges of the nuclear age.

In September 1952, Sievert and the chemist George de Hevesy, who had been at the forefront in developing radioisotopes for biological research in the 1930s in Copenhagen,^59^ had arranged a conference in Stockholm on ‘Radiobiology and Radiation Protection’.^60^ Sievert used this conference to gather a small working group of five people from the ICRP to discuss the foundation of a new organisation on radiation protection. This group met twice, on the 15th in Stockholm and on the 18th in Uppsala.^61^ Participating at the meeting were Ernest Rock Carling of the Westminster Hospital in the UK, Jaeger, Mayneord, Sievert, Taylor, and Walter Binks of the UK Radiological Protection Service. At the meeting, Sievert made the strong suggestion that the ICRP should be separated from the IRC. Key here was Sievert’s concern that the ICRP needed to expand its purview from exclusively medical matters, like formulating protection initiatives for hospitals and clinics, to also include ‘industrial hazards’ because ‘… otherwise a second International Committee might come into being to deal with radiation protection problems in the industrial field’,^62^ something he wished to avoid. Various options and approaches were discussed at the meeting, however, most were deemed impractical as the members of the ICRP ‘wished to retain as much freedom as possible’. Suggestions to nest the ICRP under a national government or another international body, for instance UNESCO, were therefore rejected, as doing so was seen as a potential jeopardy to the ICRP’s ability to freely conduct scientific research. An alternative suggestion was made that a new international body could be created to incorporate the current work of the ICRP with a view to also cover the expanding nuclear industrial sector. It was agreed that Sievert and Mayneord should prepare a note on this latter suggestion for the group’s next meeting.

At the meeting on 18 September, Sievert presented this suggestion, which had been made ‘after consultation with Professor Mayneord’.^63^ Sievert’s proposal called for a new organisation he coined ‘The International Association for Radiological Protection’ that greatly expanded the ICRP’s area of interest. In true Sievert fashion, the thorough proposal included a seven-point mission statement as well as a suggestion for the structure of the institution. This new organisation was to have a broad set of goals, including arranging conferences, ‘stimulat[ing] international co-operation’ and to facilitate the contact between relevant actors across the globe. Three main objectives were listed, aligning with Sievert’s concerns at this time: The first objective was to formulate ‘… international recommendations for radiation protection in all fields in which radiation hazards occur’. This would greatly expand the ICRP’s mission to formulate recommendations not just for medicine and radiology, but potentially all sectors that would, or could, incorporate nuclear technologies, a daunting task. The second and third objectives were encroaching on the objectives of the ICRUM, namely, that this new organisation should draw up standards and values for ‘permissible doses and dosages rates’ as well as to develop radiation units and how to measure them. The proposed organisation should also act as an ‘information bureau’, by distributing national legislation and recommendations on radiation protection to the members of the institution. Sievert’s ambition had therefore increased to not only separate the ICRP from the IRC, but also the ICRUM, and to merge these two committees into one organisation with greatly expanded mission parameters.^64^

A lengthy discussion followed at the Uppsala meeting. The group was worried about how it would be possible to financially support such a large and inclusive organisation, especially since this would require a permanent secretariat. Until this point, the secretarial work in the ICRP had been done *ad hoc* by the members of the Commission. The group discussed that while support could be sourced from organisations like UNESCO or the WHO, this would again jeopardise the principal freedom of the ICRP, as well as Sievert’s proposed new organisation. Sievert’s reply was that he would seek ‘support from private donors’. The meeting therefore ended with the members of the ICRP agreeing that, for the time being, the ICRP should not terminate its relationship with the IRC, but it should also start to widen its approach from a strict focus on medical matters so as not to allow other ‘less experienced bodies’ to encroach on this area. In addition, the group asked Sievert to prepare a final proposal for this organisation that could be circulated within the ICRP.

The next congress of the IRC and its committees was to be held in July 1953 in Copenhagen, where Sievert hoped to present a proposal for ‘an international organization for radiation protection’. A final draft for this organisation was made in late June 1953 in both an English and Swedish version.^65^ Sievert would often write first in Swedish, and then have the text professionally translated in order to appease his need for perfection.^66^ However, the reason for this two-language version was quite a different one. Sievert hoped to convince the members of the ICRP of the idea of disconnecting the ICRP and the ICRUM from the IRC by showing that it was possible to maintain a new, independent organisation through funds sourced by national foundations and groups. In order to show the feasibility of this scheme, Sievert wanted to use Sweden as a test case. Sievert’s plan was that the new IRCP-ICRUM organisation should be placed in Stockholm, arguing that Sweden would be an ideal location because of its ‘neutrality’ and good international scientific standing.^67^ A contributing factor here may also have been that this would relieve Sievert of having to undertake any travel to take part in the meetings of this organisation.

In Sievert’s proposal, he estimated that a starting capital of two million Swedish kronor would be necessary to fund the organisation and to secure a continuous budget. Sievert therefore hoped to receive 500,000 kronor through Swedish donations and the remainder through ‘foreign’ investments.^68^ Sievert accordingly circulated the Swedish-language proposal to a number of prominent actors and foundations in Sweden, among them General Consul Axel Ax:son Johnson and Harry Brynielsson of the Swedish private–public cooperation on nuclear energy, AB Atomenergi.^69^ In Sievert’s correspondence to these actors, he emphasised the potential benefits of such an organisation to Sweden and Swedish science. Swedish donations to the new organisation on radiation protection would be an investment in the future, which would allow Sweden to draw on existing strong international expertise and incorporate them domestically. The radiation protection aspect was here central, as Sievert highlighted that he and his international colleagues anxiously anticipated ‘… radiation protection problems, which we as of yet are unable to master’.^70^

Sievert’s proposal for ‘an international organization for radiation protection’ had far-reaching implications. Sievert’s eleven page proposal devoted the first three pages to explaining that while the scientific work of the ICRP and the ICRUM had been sufficient so far, the proliferation of nuclear technologies and the accompanying extension of the dangers of radiation heralded new times where: ‘… the problems of radiation protection… are no longer confined mainly to the medical use of roentgen rays and radioactive substances but have become of a more common nature in recent years’.^71^ New initiatives were therefore necessary, and Sievert formulated the aim of the organisation as being:

‘… [t]o promote research in the field of radiation protection and to direct the attention of populations and authorities to the necessity of giving sufficient consideration to injury hazards from ionizing radiations which may affect individuals, population groups, or to the whole human race.’^72^ [Underline in original text]

Focusing on research on the potential harmful effects of radiation, including genetic and other long-term issues, Sievert’s goal was not to terminate connections with the medical world, but to expand upon them. In the proposal, Sievert highlighted that: ‘Continuous contact with radiotherapy is necessary…’ especially in regards to the ‘… question of working out new treatment methods with ionizing radiations whose biological effects are still insufficiently investigated’.^73^

**Figure 2.**
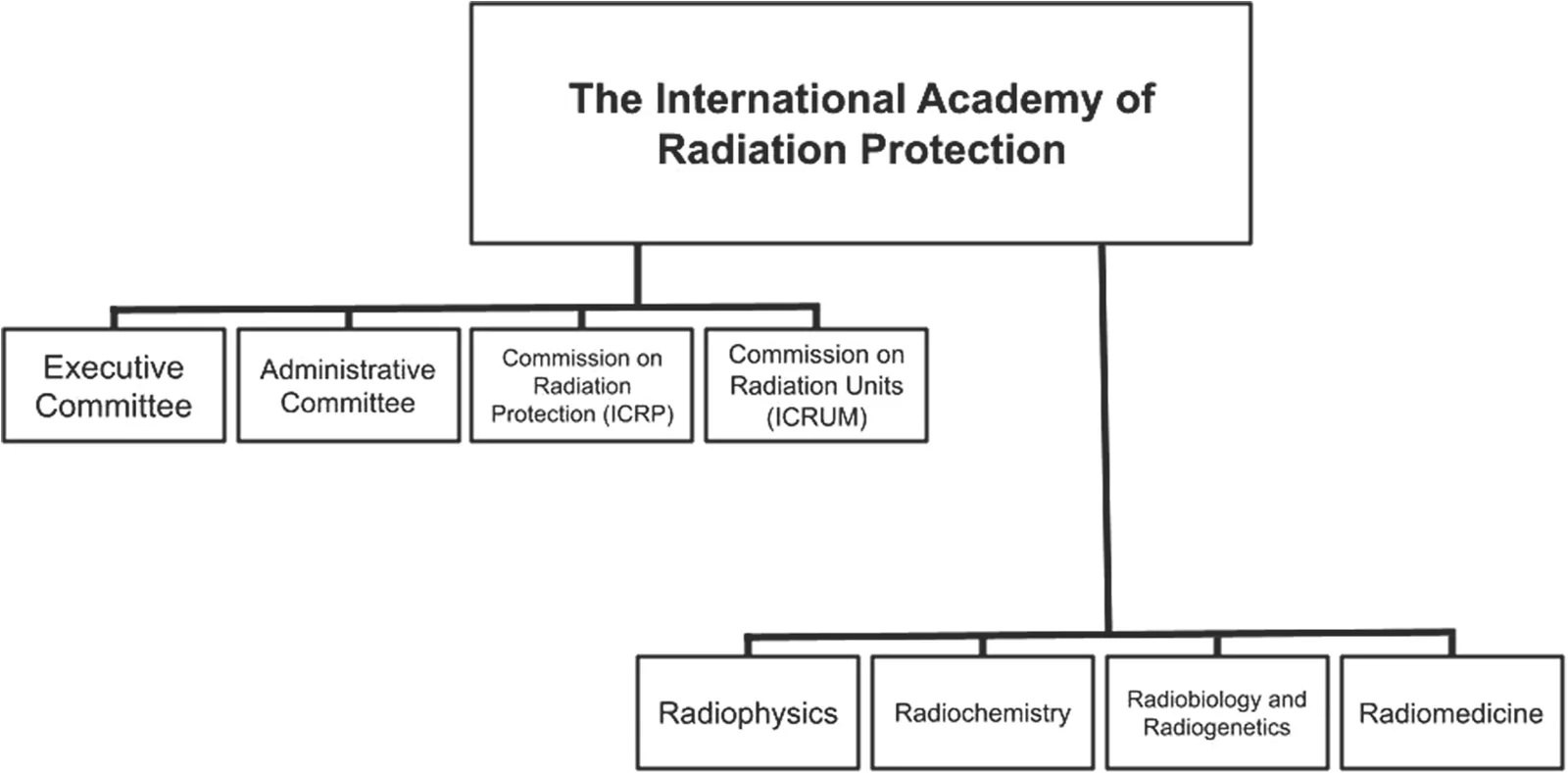
Overview of Sievert’s proposal for the ‘International Academy of Radiation Protection’ from 1953. Schematic made by the author.

Sievert imagined that the proposed organisation should be free of national interests, by only inviting the most scientifically qualified experts regardless of national affiliation. The organisation should not exceed sixty scientific members partitioned across four main sections on radiophysics, radiochemistry, radiobiology and radiogenetics, and radiomedicine. To accomplish this, the organisation should set up systems for international cooperation for actors engaged with ‘problems concerning radiation protection’ and to make quick investigations regarding radiation hazards. In addition to the information distribution function that Sievert had suggested previously, the new organisation should also ‘… facilitate the publication and distribution of scientific literature of importance for research in radiation protection and its practical application’ (Figure 2).^74^

Sievert devised a unique scheme for how to secure funding for this new organisation. The Academy should consist of a principal secretariat, with several sub-secretariats to be set up in the countries that had contributed financial support to the organisation, which Sievert at first identified as West Germany, Great Britain, France, Sweden, and the USA. The administrative committee of the organisation should reflect this scheme by including members of the countries that financially contributed to the Academy. It seems somewhat paradoxical that Sievert imaged a selection process for the scientific members of the Academy that should forgo any national biases, while at the same time using a financial scheme that was privileging national financial contributors.

While this new organisation was considerably more expansive in several key aspects than its ICRP and ICRUM counterparts, its function was not. Sievert highlighted that it was not the aim of this organisation to be a regulatory or statutory institution; instead, the Academy should focus on ‘making suggestions and preparing recommendations for the guidance of national bodies’.^75^

This new Academy should be viewed as an expression of a number of factors related to Sievert’s preferences and experiences in three areas. Firstly, the strong emphasis on the economic, political, and institutional independence of the proposed organisation should be attributed to Sievert’s poor experiences with the LoN in the inter-war period. The encounter with the LoN represented a great frustration for Sievert, and encouraged him to avoid incorporating his proposed organisation in an existing UN body, like the WHO or UNESCO. For Sievert, it was necessary to carve out an area in the international landscape where an independent ICRP was to be *the* scientific authority on radiation protection. Secondly, Sievert’s aversion to larger committees with many members impacted the membership and selection process of his new organisation. It is telling that the individual sections of Sievert’s 1953 proposal would be represented at most with two members at the organisation’s executive committee, meaning that no one section would be able to overwhelm the other groups like the USA, UK, and Canadian grouping had done to Sievert at the 1950 IRC.

Thirdly was Sievert’s concern for the dangers he foresaw with the advent of nuclear technologies after 1945. Sievert had been concerned with the issues of small dosages of radiation and their effects on humans since at least the 1930s,^76^ however, the rapid expansion of nuclear power, radioisotopes, and atomic weapons testing meant that the question was becoming more pressing. If the industrial uses of nuclear proliferated at an exponential rate, along with the radioactive fallout following nuclear bombing testing, the risk of a general, global increase of radiation exposure for all humans presented public health issues of a previously unknown size and dimension. Sievert also expressed this concern at his opening speech at the IRC in Copenhagen in July 1953, stating that: ‘With the advances in nuclear physics, we are entering a period when radiation hazards will in all probability become a general problem of rapidly increasing importance in all civilized countries’.^77^ Sievert foresaw that ‘… a great many fundamental problems in these fields are still unsolved’.^78^ The issues of thresholds and permissible dosages of radiation exposure were problematic, as it was unknown what effects a chronic, low dosage of radiation would mean for human beings. Indeed, Sievert argued it was prudent to be ‘extremely cautious with ionizing radiations’, since the negative consequences of radiation did not appear instantly and that the accumulation of radiation over a longer period of time could have fatal results.^79^ Most worryingly, Sievert believed that the current authorities on radiation protection were ill-equipped to deal with this new reality.^80^ It was therefore necessary to found an organisation that could act quickly and adapt to the problems of the atomic age as they arose, independent of any political or institutional bias, to mitigate the harmful effects of ionising radiation.^81^

## A new ICRP, 1954–1960^82^

Despite Sievert’s efforts, his proposal for the new organisation was not successful at the 1953 IRC. Sievert’s ambition of securing funding through donors in Sweden did not bear fruit, and while the proposal was discussed at the Copenhagen Congress, it was once again rejected, keeping the ICRP as part of the IRC for the time being.^83^ These setbacks did not deter Sievert, and he would continue throughout the 1950s to establish an organisation that could deal with the challenges of the nuclear age.

In 1956, Sievert took over the chairmanship of the ICRP, and in that same year, put forward a revised proposal for the radiation protection organisation, which he hoped to secure funding for this time through the Ford Foundation.^84^ Notably, Sievert’s focus on dissemination of information and literature had expanded: the new organisation should publish a journal with the title *International Journal of Radiation Protection* as well as a handbook on recommendations and other materials. In addition, the organisation should set up a global system of measurements of nuclear fallout and background radiation. According to Lindell, Sievert hoped to take over roles already partitioned by UNESCO and WHO on radiation protection with this new organisation.^85^ However, this suggestion would also ultimately fail, as Sievert was once again not able to secure funding.

**Figure 3.**
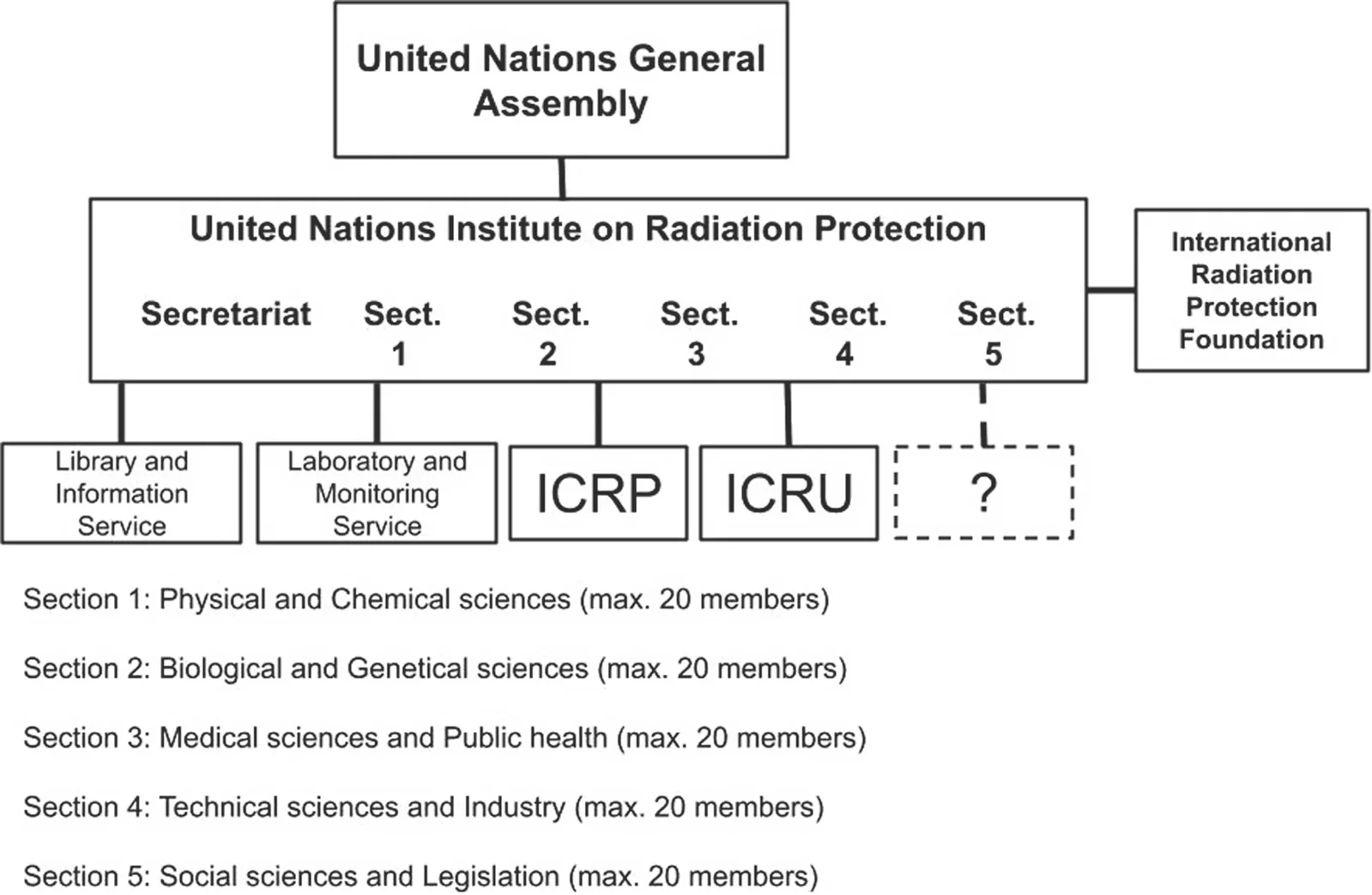
Sievert’s 1958 proposal for a radiation protection organization. Taken from Taylor, page 8:463 and adapted into this schematic made by the author. Figure 3. Long description.

Towards the end of the 1950s, following a string of failed attempts, Sievert saw no other option than nesting his new organisation on radiation protection and measurement within the UN-family, in order to secure a continuous budget for the group’s operation. Sievert seems to have come to this conclusion also based on the foundation of the United Nations Scientific Committee on the Effects of Atomic Radiation (UNSCEAR) in 1955^86^ and the IAEA in 1957,^87^ both of which would become key in Sievert’s further plans.^88^ In April 1958, Sievert sent his newest proposal to Taylor, hoping to secure his support for the new radiation protection organisation. The 1958 proposal was similar to the 1953 and 1956 suggestions, however, it differed in two key areas (Figure 3). Firstly, Sievert imagined that this new organisation should also conduct independent research through the foundation of a research laboratory for the ‘…investigation of radioactive samples, developments of monitoring methods and standardization of equipment for these purposes’.^89^ In addition, the organisation should maintain an independent ‘international monitoring service’ for radioactivity.^90^ The second key area was the organisation of the group. In his proposal, Sievert highlighted two options: either to keep the ICRP, the ICRUM, and the UNSCEAR as independent organisations, or, more radically, to combine these three organisations into one specialised agency on radiation protection within the UN.^91^ Sievert’s preference was naturally for the latter option, highlighting that this new group would need an independent and ‘flexible’ structure that would allow it to manoeuvre in cases of nuclear catastrophes. Funding for this scheme would be secured through a new foundation with several national and business sponsors, ensuring total financial independence.^92^

Taylor was sceptical of Sievert’s suggestion, arguing that keeping the ICRP and the ICRU as independent groups under the IRC seemed the preferable option to him. The second alternative, the creation of a new group under the UN, Taylor considered undesirable, as it would:

‘… merely extend the already enormous confusion that exists between the various members of the UN family. Here we already have the UNSC [UNSCEAR], WHO, UNESCO, and the IAEA, all struggling for priority in the field of radiation protection. I do not believe that the establishment of another organization would do anything to simplify this.’^93^

Especially the IAEA seemed to present an obstacle to Sievert’s proposed organisation. Sievert’s proposal to create an independent research laboratory along with the monitoring of radioactivity was highlighted by Taylor as primary interests of the IAEA, and, as such, it would be difficult for other organisations to take over these responsibilities.^94^ It is easy to see why Taylor thought this. The IAEA had ongoing plans to build a radiation dosimetry laboratory since 1957, culminating in the Seibersdorf Laboratory in 1961 near Vienna.^95^ At the same time in 1958 and 1959, the IAEA was in active negotiations with the WMO regarding global monitoring services of radioactivity, which the IAEA was vying to have primacy in.^96^ Both of these areas would come into direct conflict with Sievert’s new organisation. In his reply, Taylor stated that he approved of the majority of Sievert’s proposal, but found these critical points untenable. Sievert countered, stressing the need for change and for the ICRP and the ICRUM to be re-oriented and to expand their operations: ‘If we [Sievert and Taylor, the ICRP and ICRUM] continue on the present scale of our work I am sure that will soon lose our reputation because we have not sufficiently realised the new order of importance of our task.’ ^97^ [Underline in original] Sievert further insisted that the situation was so dire that nesting the new radiation protection organisation under the UN was ‘our last chance’.

Towards the end of 1959 and early 1960, Sievert would finally find success in his endeavours. However, this was in a much less spectacular fashion than he had imagined. These successes came primarily within three main areas. First, through negotiations in 1960, Sievert had managed to secure the ICRP an annual grant for five years through the Ford Foundation in the area of 70,000 dollars.^98^ In comparison, Sievert’s 1958 proposal for an independent ICRP had asked for ten million dollars with yearly contributions around the one million mark.^99^ However, while modest, the 70,000-dollar grant secured the independent operations of the ICRP for the foreseeable future. Secondly, as highlighted, Sievert had attempted to introduce an independent handbook for radiation protection measurements and technical details since at least 1956, and he had already been interested in the dissemination of information since 1952. Previously, the ICRP recommendations had always been published as a supplement in national radiological journals, which Sievert considered to be disadvantageous as it took away from the international character of the ICRP.^100^ Sievert had attempted to publish the ICRP handbooks through a Swedish publisher, however, a chance encounter between Failla and the founder of Pergamon Press in 1959 led to the ICRP’s recommendations being published through them.^101^ Third was Sievert’s insistence that the ICRP should expand its area of interest from medical and radiological work to other, general populations. This was achieved in 1957 in a back-door fashion by Lindell and Sievert. For various reasons, it had proved difficult to prepare the ICRP’s full recommendations in time for publication in 1957 as had been planned. As a consequence, Lindell had suggested to Sievert that they publish a short amendment in several radiological journals, which included a small but decisive new provision specificity that the ICRP’s recommendations covered the genetic effects on *all* populations affected by radiation exposure, and not just workers.^102^ In effect, the ICRP had thereby expanded its purview from only medical personnel to cover all humans affected by radiation, meaning in practice all of humanity.

## Conclusion

Rolf Sievert, as a diplo-scientific actor, was instrumental in shaping the trajectory of the ICRP in the post-war period, and thereby also the international landscape of radiation protection and regulation. While it has never been the stated objective of the ICRP to perform regulatory action, several national, regional, and international bodies within different professional fields use the recommendations of the ICRP as the basis for their safety standards and initiatives.^103^ These recommendations therefore inform work with radioactivity and at radioactive sites, as well as epistemic claims of knowledge about the biological and medical effects of radiation. However, the current state of the ICRP as a body engaged with making safety recommendations on radioactivity for all populations was primarily a result of Sievert’s engagement, negotiations, and diplomatic compromises in the 1950s.

As a member of the ICRUM and founding president of the ICRP, Sievert had been engaged within the international field of radiation standardisation and safety recommendations since the mid-1920s. Following the debates between the USA and UK delegations in 1937, the scientific upheaval on the issues of radiation standardisation meant that Sievert was convinced that new action had to be taken in order to unify on a new radiation standard that could be used to conduct proper scientific work and inform clinical practice. The invitation to the League of Nations’ Groningen conference represented such an opportunity. However, Sievert found the League of Nations’ system cumbersome and inflexible, preventing efficient action. With the atomic bombings of Hiroshima and Nagasaki, a new reality set in foreboding a future where not just medical personnel and patients would be affected by exposure to radiation but potentially all of humanity. Sievert was deeply concerned about this state-of-affairs, and was simultaneously convinced that the ICRP would not be able to fully handle the challenges of the atomic age unless changes were carried out. He therefore set out to divorce the ICRP from the IRC, to form a new agile organisation that would be independent of outside interests and able to meet the challenges of the atomic age head-on. However, securing support within the ICRP and the ICRUM proved difficult, and Sievert was unable to solve the issue of finding funding for this new organisation.

While Sievert never completely gave up his plans for an independent radiation protection group, by 1960, the need for a new organisation was diminished through the initiatives he had successfully established through the ICRP. Through Sievert’s actions, the ICRP remained an international, scientific group independent of outsider interests, that regularly disseminated information on radiation protection, no longer just for radiologists and medical patients but for *all* populations. This also extended the role of the ICRP, radiation protection, and nuclear research into medicine from a relatively specialised medical sub-domain to an area of global concern. While the new organisation that Sievert had imagined never came to be, a majority of its goals were still carried out through a re-configured ICRP that continues to shape the international landscape of radiation protection.

Sievert began his reconfiguration attempts because of his personal concerns, and tried to mould the new organisation to fit his preferences. Key here was also Sievert’s diplomatic negotiations and considerations; Sievert continuously adapted his proposal based on what he believed he could accomplish within the contemporary landscape of international organisations, even attempting to take over areas from other groups, for instance, the dosimetry laboratory of the IAEA or the monitoring network of the WMO. However, Sievert’s negotiations were not just international, but also domestic. In his attempts to secure donations from Swedish private donors in 1953, Sievert highlighted how international knowledge could benefit Swedish national interests, with his proposed organisation even being based in Stockholm. While Sievert’s proposals were made to fit within his own overarching wishes and ideals, they were also adaptable to suit the international landscape at the time and the financial actors that Sievert attempted to court, combining Sievert’s scientific interests with his diplomatic activities.

With this article, I have aimed to show the benefit of focusing on specific *diplo-scientific actors* as important figures in shaping international organisations and their standardisation efforts. Previous research has highlighted how scientists have played a central role in the standardisation of units and measurements through social processes, which also reveal that standardisation is not a neutral process, but deeply interwoven with politics, governance, and commercial interests.^104^ This is particularly the case for international organisations, which have come to be some of the primarily arenas of scientific standard setting also in a diplomatic fashion.^105^ International organisations are doubly important here, as they also act as mediators in structuring scientific and medical practices in local, regional, and national contexts. By standardising methods, instruments, policies, and epistemic claims of knowledge and scientific consensus, international organisations help to shape the modern landscape of scientific practice.^106^ However, international organisations are not neutral entities, rather, they are created through scientific and diplomatic practices and activities carried out by the actors – like Sievert – who helped found and develop them. So far, however, these diplo-scientific actors have not been thoroughly studied within the science diplomacy scholarship.^107^ Highlighting the case of Sievert and the ICRP, I hope to have added to the science diplomacy literature with an assessment of how the interconnected and co-produced aspects of science and diplomacy carried out by specific actors can shape the development of international organisations and their standardisation efforts. Sievert is here illustrative, as it is likely that many of the current activities of the ICRP would not have come into being without Sievert’s attempts to divorce the Committee from the IRC in the 1950s. Sievert, as a diplo-scientific actor, therefore highlights the importance of also considering the impact of individual actors in international relations and organisations in further research.

## Footnotes

1 In the 1920s in the USA, several young female factory workers developed horrible illnesses as a result of their work painting watch dials with radium paint. These women were dubbed the ‘radium girls’, and were part of one of the first public controversies involving radiation. For literature on the Radium Girls, see Claudia Clark , Radium Girls: Women and Industrial Health Reform, 1910–1935 (Chapel Hill: University of North Carolina Press, 1997)11623540; Kate Moore , The Radium Girls: The Dark Story of America’s Shining Women (Naperville: Sourcebooks, Inc., 2017). For radiation protection prior to 1939, see Daniel Paul Serwer , The Rise of Radiation Protection: Science, Medicine and Technology in Society, 1896–1935 (Brookhaven National Laboratory, 1976); Bo Lindell , Pandora’s Box: The Time Before World War II (Nordic Society for Radiation Protection, 2019); J. Samuel Walker , Permissible Dose: A History of Radiation Protection in the Twentieth Century (Berkely: University of California Press, 2000). For the blurred lines between fraud and medical treatment, see Maria Rentetzi , Seduced by Radium: How Industry Transformed Science in the American Marketplace (Pittsburgh: University of Pittsburgh Press, 2022).

2 Walker, 2000, *op. cit.* (note 1), 11; Rebecca S. Lowen , ‘Entering the Atomic Power Race: Science, Industry and Government’, Political Science Quarterly, 102, 3 (1987), 461 and 476; Jacalyn Duffin and Charles R. R. Hayter , ‘Baring the Sole: The Rise and Fall of the Shoe-Fitting Fluoroscope’, Isis, 91, 2 (2000), 278–7910.1086/38472110967875.

3

Carolyn Kopp , ‘The Origins of the American Scientific Debate over Fallout Hazards’, Social Studies of Science, 9 (1979), 403–2210.1177/03063127790090040211610816.

4

K.V. Khanna Sansare and F. Karjodkar , ‘Early Victims of X-rays: a Tribute and Current Perception’, Dentomaxillofacial Radiology, 40 (2011), 123–2510.1259/dmfr/73488299PMC352029821239576; J. Zakovsky , ‘Ergänzung und Einbau der Empfehlungen für Strahlenschutz in Internationale Strahlenschutzvorschriften’, Acta Radiologica, 42, 116–supplement (1954), 32-33 13228159. Several prominent scientific actors had also perished following overexposure, most notably Marie Curie in 1934.

5

Aske Hennelund Nielsen and Maria Rentetzi . ‘The Search for Radiation Standards and Science Diplomacy in the Interwar Period’, Physics in Perspective, 26 (2024), 237–267 ; Maria Rentetzi and Aske Hennelund Nielsen , ‘Setting Radiation Protection Standards: The First Radiology Congress in 1925’, Medical Research Archives, 12, 6 (2024), 1–7 .

6 See Ito Kenji and Maria Rentetzi , ‘The Co-production of Nuclear Science and Diplomacy: Towards a Transnational Understanding of Nuclear Things’, History and Technology, 37, 1 (2021), 4–20 ; Matthew Adamson and Roberto Lalli , ‘Global Perspectives on Science Diplomacy: Exploring the Diplomacy Knowledge Nexus in Contemporary Histories of Science’, Centaurus, 63 (2021), 1–16 ; Simone Turchetti , Matthew Adamson , Giulia Rispoli , Doubravka Olšáková and Sam Robinson . ‘Just Needham to Nixon? On Writing the History of “Science Diplomacy”’, Historical Studies in the Natural Sciences, 50, 4 (2020): 323–39.

7

Henry Nielsen and Henrik Knudsen , ‘The Troublesome Life of Peaceful Atoms in Denmark’, History and Technology, 26, 2 (2010), 91–118 .

8

Lawrence Busch , Standards: Recipes for Reality (Cambridge: The MIT Press, 2011), 2 and 13.

9 For instance: Sverker Sörlin , ‘The Anxieties of a Science Diplomat: Field Coproduction of Climate Knowledge and the Rise and Fall of Hans Ahlmann’s “Polar Warming”’, Osiris, 26, 1 (2011), 66–88 10.1086/66126521936187; Finn Aaserud , ‘Niels Bohr’s Diplomatic Mission during and after World War Two’, Berichte zur Wissenschaftsgeschichte, 43 (2020), 493–520 10.1002/bewi.20200002633283305; Finn Aaserud , ‘The Scientist and the Statesmen: Niels Bohr’s Political Crusade during World War II’. Historical Studies in the Physical and Biological Sciences, 30, 1 (1999), 1–47 .

10

Bruno Latour and Steve Woolgar , Laboratory Life: The Construction of Scientific Facts (New Jersey: Princeton University Press, 1986), 21–22 , 31–32, and 237.

11

Dominique Pestre , ‘Science, Political Power and the State’ in John Krige and Dominique Pestre (eds), Companion to Science in the Twentieth Century (New York: Routledge, 2003), 63–64 .

12

Charlotte Bigg , Jessica Reinisch , Geert Somsen and Sven Widmalm , ‘The Art of Gathering: Histories of International Scientific Conferences’, The British Journal for the History of Science, 56 (2023), 429–431 10.1017/S000708742300063837902418.

13 Late in the writing of this article, the article ‘Over Spilt Milk’ by Alma Igra was published, which also includes discussions of ‘scientist-diplomats’ (Igra’s term) and their role in shaping international standards. See Alma Igra , ‘Over Spilt Milk: British Scientific Humanitarianism and the Quest for International Standards’, Isis, 115, 2 (2024): 335–53.

14 See for instance Helge Kragh , ‘On Scientific Biography and Biographies of Scientists’ in T. Arabatzis , J. Renn and A. Simões (eds), Relocating the History of Science (Springer, 2015), 269–80; Mary Jo Nye , ‘Biography and the History of Science’ in T. Arabatzis , J. Renn and A. Simões (eds), Relocating the History of Science (Springer, 2015), 281–96; Mary Jo Nye , ‘Scientific Biography: History of Science by Another Means?’, Isis, 97, 2 (2006), 322–29; Mott T. Greene , ‘Writing Scientific Biography’, Journal of the History of Biology, 40, 4 (2007), 727–59.

15 For literature on Sievert, see Hans Weinberger, *Sievert: enhet och mångfald* (Stockholm: KTH, 1990) for a comprehensive Swedish biography that has been invaluable for this article; Bo Lindell, Sievert’s long-time colleague and secretary of the ICRP, and his series on radiation protection, in particular *The Labours of Hercules, 1950–1966* (Nordic Society for Radiation Protection, 2020); Sekiya and Yamasaki have made an overview of Sievert’s life and accomplishments based on Weinberger’s book: Masaru Sekiya and Michio Yamasaki ‘Rolf Maximilian Sievert (1896–1966): Father of Radiation Protection’, Radiological Physics and Technology, 9, 1 (2016)10.1007/s12194-015-0330-526220621.

16 Weinberger, *op. cit.* (note 15), 167–68.

17
*Ibid.*, 166. The Sievert unit (abbreviated as Sv) is a biological unit that represents the health effects of ionising radiation on humans. This type of biological unit differs from previous units used in radiology before the mid-1950s, that were purely physical units, that is to say, the units were only meant to measure how much radiation was present in a given context, and not the health effects on the patient or organism.

18
*Ibid.*, 161.

19

Rolf M. Sievert , ‘Untersuchungen über die an verschiedenen Schwedischen Krankenhäusern zur Erreichung des Hauterythems Gebräuchlichen Röntgenstrahlenmengen, unter Einführung der »R»-Einheit’, Acta Radiologica 7, 1-6 (1926), 463 and 472.

20 See Angela N. H. Creager , Life Atomic: A History of Radioisotopes in Science and Medicine (Chicago: University of Chicago Press, 2015).

21 Originally named the International X-Ray Unit Committee (IXUC) but renamed in 1931 to the ICRUM. For the sake of clarity, I use the current name and abbreviation.

22 Like the ICRUM, the current name of ICRP will be used here. The group was originally founded under the name the International X-Ray and Radium Protection Committee (IXRPC).

23 See R. H. Clarke and J. Valentin , ‘The History of the ICRP and the Evolution of its Policies’ Annals of the ICRP, 39 (2009), 75–110 .

24 Hennelund Nielsen and Rentetzi, ‘The Search for Radiation Standards’, *op. cit.* (note 5). See also Serwer *op. cit.* (note 1), 205–08.

25 Letter from Taylor to Sievert (8. April 1937) in Karolinska Institute (Stockholm): Institutionen för radiofysik 1924–1965. Reference code: SE/49:2. Series F1, Package 84, File V-D 4a [Hereafter ‘Karolinska’]

26 I have not been successful in finding any biographical data for Establier.

27 Letter from Establier to Sievert (6 September 1938) in Karolinska. F1. Package 84. File V-D 4b.

28 The full list of invitees was: Albert Bouwers of the Philips Naturkundig Laboratorium in the Netherlands, Failla, the German physicist Walter Friedrich, G. Garcia, Gray, Holthusen, the French Physicist Fernand Holweck, Robert Jaeger of the Physikalische-Technischen Reichsanstalt in Berlin, Antoine Lacassagne of the Pasteur Laboratory at the Radium Institute in Paris, the French radiologist Lucien Mallet, Mayneord and J. Murdoch of Brussels University. In his letter, Establier only listed the first initials and full last name of the radiologists in the order given here. I have been unsuccessful in finding any information on Garcia.

29 Lindell, *op. cit.* (note 1), 131. For the history of the League of Nations’ technical conferences, see Jessica Reinisch , ‘Technical Conferences as a Technique of Internationalism’, The British Journal for the History of Science, 56 (2023), 485–502 10.1017/S000708742300033X37697659.

30 For instance, according to *The Lancet*, while the report by the two German scientists ignored the ‘pioneering’ work of the UK on radiation protection, the qualities of the report were obvious: ‘… it would be ungenerous not to bestow high praise for its thorough and critical treatment of the main issues’. ‘X Ray Protection’, *The Lancet*, 219, 5660 (1932), 408.

31

Stanley W. Pycior , ‘“Her Only Infidelity to Scientific Research”: Marie Skłodowska Curie and the League of Nations’, The Polish Review, 41, 4 (1996), 449 footnote 2.

32 Letter from Holthusen to Sievert (17 September 1938) in Karolinska. F1. Package 84. File V-D 4b.

33 The duality of diplo-scientific actors being both independent scientific experts who also represent national interests, is a general dilemma that has not received enough scholarly attention. See also Bigg *op. cit.* (note 12), 430.

34 Letter from Holthusen to Sievert (17 September 1938) in Karolinska. F1. Package 84. File V-D 4b. My translation.

35 Letter from Sievert to Holthusen (23 September 1938) in Karolinska. F1. Package 84. File V-D 4b.

36 Weinberger, *op. cit.* (note 15), 56.

37 Lindell, *op. cit.* (note 1), 128.

38 Letter from Sievert to Holthusen (23 September 1938) in Karolinska. F1. Package 84. File V-D 4b.

39 Letter from Sievert to Establier (23 September 1938) in Karolinska. F1. Package 84. File V-D 4b

40 Letter from Establier to Sievert (3 October 1938) in Karolinska. F1. Package 84. File V-D 4b

41 Letter from Sievert to Establier (12 October 1938) in Karolinska. F1. Package 84. File V-D 4b

42 Weinberger, *op. cit.* (note 15), 135; Lindell, *op. cit.* (note 15), 179.

43 Lindell, *op. cit.* (note 15), 86.

44 Weinberger, *op. cit.* (note 15), 42.

45 Letter from Failla to Sievert (5 May 1939) in Karolinska. F1. Package 84. File V-D 4b.

46 ‘Edith Hinkley Quimby’, *The Journal of Nuclear Medicine*, 6, 5 (1965), 383–85.

47 Letter from Sievert to Establier (16 May 1939) in Karolinska. F1. Package 84. File V-D 4b.

48 Letter from Sievert to Establier (26 May 1939) in Karolinska. F1. Package 84. File V-D 4b.

49

Paul N. Edwards , ‘Meteorology as Infrastructural Globalism’, Osiris, 21, 1 (2006), 235 .

50

Ana Barahona , ‘Radiation Risk in Cold War Mexico: Local and Global Networks’, NTM Zeitschrift Für Geschichte der Wissenschaften, Technik und Medizin, 30, 2 (2022), 246–247 10.1007/s00048-022-00331-0PMC916012935536307.

51

Soraya Boudia , ‘Global Regulation: Controlling and Accepting Radioactivity Risks’, History and Technology, 23, 4 (2007), 389 , quote from 400.

52 For the history of the crowded post-war landscape of international organisations, see also Jenny Beckmann , ‘Niche Development: the International Foundation for Science and the Road to Sweden’, The British Journal for the History of Science, 56 (2023), 533–566 10.1017/S000708742300054737861071.

53 Maria Rentetzi, ‘The Politics of Radiation Protection’, *NTM Zeitschrift für Geschichte der Wissenschaften, Technik und Medizin*, 30, 2 (2022), 125–35; Maria Rentetzi, ‘The Global Experiment: How the International Atomic Energy Agency Proved Dosimetry to Be a Techno-Diplomatic Issue’, *NTM Zeitschrift für Geschichte der Wissenschaften, Technik und Medizin*, 30, 2 (2022), 168–69; Linda Marie Richards, ‘1945–1964 WHO’s Right to Health?’ *NTM Zeitschrift für Geschichte der Wissenschaften, Technik und Medizin*, 30, 2 (2022), 138; Aske Hennelund Nielsen, ‘A Global Nuclear Environmental Infrastructure: The World Meteorological Organization and the International Atomic Energy Agency’s Global Fallout Monitoring Program in the 1960s’, paper given at the ‘European Society for Environmental History Conference’ Bern, Switzerland (22 to 26 August 2023).

54 Lindell, *op. cit.* (note 15), 86.

55
*Ibid.*, 87.

56
*Ibid.*

57 ‘International Recommendations on Radiological Protection’, *The British Journal of Radiology*, 24, 277 (1951), 46–53.

58
*Ibid.*, 46 and 50.

59 For Hevesy, see Hilde Levi , George de Hevesy: Life and Work: a Biography (Copenhagen: Rhodos, 1985); Siegfried Niese , Georg von Hevesy 1885–1966 (Münster: Principal, 2009).

60

George de Hevesy and Rolf Sievert , ‘Conference on Radiobiology and Radiation Protection Stockholm’ Acta Radiologica, 41, 1 (1954), 1 .

61 ‘International Commission on Radiological Protection’ and ‘Meeting on 18th September 1952, in Uppsala’ in Karolinska. F2. Package 1.

62 ‘International Commission on Radiological Protection’ in Karolinska. F2. Package 1.

63 ‘Meeting on 18th September 1952, in Uppsala’ in Karolinska. F2. Package 1.

64 Lindell, *op. cit.* (note 15), 97; Weinberger, *op. cit.* (note 15), 141.

65 ‘Suggestion for an international organization for radiation protection’ and ‘Förslag till en internationell organisation för strålskydd’ (both dated 30 June 1953) in Karolinska. F2. Package 1.

66 Weinberger, *op. cit.* (note 15), 162. There are numerous examples of this in the Karolinska Archives as well.

67 ‘P. M. angående behovet av kapital för startandet av en internationelle strålskyddsorganisation’ (dated 15. June 1953) in Karolinska. F2. Package 1. See also Weinberger, *op. cit.* (note 15), 145. On science and neutrality in Sweden see Sven Widmalm , ‘“A Superior Type of Universal Civilisation”: Science as Politics in Sweden, 1917–1926’ in Rebecka Lettevall , Geert Somsen , and Sven Widmalm (eds), Neutrality in Twentieth-Century Europe (New York: Routledge, 2012), 65–89 .

68 ‘P. M. angående behovet av kapital för startandet av en internationelle strålskyddsorganisation’ (dated 15 June 1953) in Karolinska. F2. Package 1.

69 Letter from Sievert to Axel Axelson Johnson (9 July 1953) in Karolinska. F2. Package 1. My translation. On the history of nuclear energy and AB Atomenergi in Sweden, see Karl-Erik Larsson , ‘Kärnkraftens historia i Sverige’, Kosmos, 64 (1987), 121–61.

70 Letter from Sievert to Albin Johansson (9 July 1953) in Karolinska. F2. Package 1. My translation.

71 ‘Suggestion for an international organization for radiation protection’ (dated 30 June 1953) in Karolinska. F2. Package 1.

72
*Ibid.*

73
*Ibid.*

74
*Ibid.*

75
*Ibid.*

76 ‘Redogörelse för undersökningar av i handeln förekommande, svagt radioaktiva preparat utförda vid Radiumhemmets fysiska laboratorium’ in Karolinska. F1. Package 84.

77

Rolf M. Sievert , ‘Viewpoints on the Organization of Radiation Protection’, Acta Radiologica, 24, 116–supplement (1954), 3613228160.

78
*Ibid.*, 35.

79
*Ibid.*, 35–36.

80
*Ibid.*, 38.

81
*Ibid.*, 38–39.

82 Post the 1953 IRC, the archival materials related to Sievert’s attempts to re-orient the ICRP became limited. According to Taylor, from the second half of the 1950s, Sievert avoided leaving a paper trail of his activities. Despite this, Sievert’s efforts have been documented in other sources which inform his section of the article.

Lauriston S. Taylor , Organization for Radiation Protection: The Operations of the ICRP and NCRP 1928–1974 (Springfield: National Technical Information Service, 1979), 8:449 .

83 Lindell, *op. cit.* (note 15), 96.

84
*Ibid.*,183.

85
*Ibid.*,183–184.

86 Sievert served as the Swedish representative at the creation of UNSCEAR along with representatives from 14 other states. Sievert’s role in forging UNSCEAR seems to have been quite substantial, with some sources calling him the ‘godfather’ of the group ( Ray Appleyard , ‘The Birth of UNSCEAR—The Midwife’s Tale’, Journal of Radiological Protection, 30, 3 (2010), 621 10.1088/0952-4746/30/3/M0120826894) and others simply the ‘Father of the Committee [UNSCEAR]’ ( David Sowby , ‘Some Recollections of UNSCEAR’, Journal of Radiological Protection, 28, 3 (2008), 274 10.1088/0952-4746/28/3/E0318716348). To my knowledge, no study has looked specifically at Sievert’s contribution to the creation of UNSCEAR. For the history of UNSCEAR, see Néstor Herran , ‘“Unscare” and Conceal: The United Nations Scientific Committee on the Effects of Atomic Radiation and the Origin of International Radiation Monitoring’ in Simone Turchetti and Peder Roberts (eds), The Surveillance Imperative: Geosciences during the Cold War and Beyond, (New York: Palgrave Macmillan, 2014), 69–84 .

87 Taylor, *op. cit.* (note 82), 8:451.

88
*Ibid.*, 8:458.

89
*Ibid.*, 8:459.

90
*Ibid.*, 8:459 and 8:461.

91
*Ibid.*, 8:460.

92
*Ibid.*

93
*Ibid.*, 8:449.

94
*Ibid.*

95 Rentetzi, ‘The Global Experiment’, *op. cit.* (note 53), 172 and 174–75.

96 Hennelund Nielsen, *op. cit.* (note 53).

97 Taylor, *op. cit.* (note 82), 8:466.

98 Lindell, *op. cit.* (note 15), 234.

99 Taylor, *op. cit.* (note 82), 8:462.

100 Lindell, *op. cit.* (note 15), 231.

101
*Ibid.*

102
*Ibid.*, 210–211. The recommendations were published in several journals, for instance in *Acta Radiologica* which Lindell also mentions. See ER. Carling , W. Binks , L. Bugnard , et al., ‘Report on Amendments During 1956 to the Recommendations of the International Commission on Radiological Protection’, Acta Radiologica, 48, 6 (1957), 493–95.

103 ICRP ‘Application of the Commission’s Recommendations for the Protection of People in Emergency Exposure Situations’, *ICRP Publication*, 109 (2009), 102 figure 3.1.

104

Theodore M. Porter , Trust in Numbers: The Pursuit of Objectivity in Science and Public Life (New Jersey: Princeton University Press, 1995), 11–12 and 27; M. Norton , Wise ‘Introduction’, in M. Norton Wise (ed.), The Values of Precision (New Jersey: Princeton University Press, 1995), 5–6 ; Simon Schaffer , ‘Accurate Measurement is an English Science’, in M. Norton Wise (ed.), The Values of Precision (New Jersey: Princeton University Press, 1995), 135–137 .

105 Bigg *op. cit.* (note 12), 428 and 430.

106 For general methodological considerations on how international organisations affect underlaying and participating national, regional, and local groups, see Wolfram Kaiser and Jan-Henrik Meyer , ‘Conclusion: Setting Agendas, Building Institutions and Shaping Binding International Commitments’, in Wolfram Kaiser and Jan-Henrik Meyer (eds), International Organizations and Environmental Protection (New York: Berghahn Books, 2016), 317–32; Ivan Lind Christensen and Christian Ydesen , ‘Routes of Knowledge: Toward a Methodological Framework for Tracing the Historical Impact of International Organizations’, European Education, 47, 3 (2015), 274–88. For an example of international organisations affecting national science, see Edwards *op. cit.* (note 49), 239–41.

107 With some notable exceptions being Igra, *op. cit.* (note 13) and Gisela Mateos and Edna Suárez-Díaz , ‘Atomic Ambassadors: the IAEA’s First Preliminary Assistance Mission (1958)’, History and Technology 37, 1 (2021), 90–105 .

## Long descriptions

### Figure 3. Long description

The flowchart begins at the top with a box labeled United Nations General Assembly. A vertical line connects downward to a wide box titled United Nations Institute on Radiation Protection. To the right of this institute box, a horizontal line connects to an external box labeled International Radiation Protection Foundation.

Inside the United Nations Institute on Radiation Protection box, there is a Secretariat and five numbered sections: Sect. 1, Sect. 2, Sect. 3, Sect. 4, and Sect. 5.

Below the institute box, vertical lines connect to five specific entities:

- Below Secretariat is the Library and Information Service.

- Below Sect. 1 is the Laboratory and Monitoring Service.

- Below Sect. 2 and Sect. 3 is I C R P.

- Below Sect. 4 is I C R U.

- Below Sect. 5 is a dashed box containing a question mark.

At the bottom of the diagram, a text legend defines the five sections:

- Section 1: Physical and Chemical sciences (max. 20 members)

- Section 2: Biological and Genetical sciences (max. 20 members)

- Section 3: Medical sciences and Public health (max. 20 members)

- Section 4: Technical sciences and Industry (max. 20 members)

- Section 5: Social sciences and Legislation (max. 20 members)

Navigate back to Figure 3..

